# Lung Cryobiopsy for the Diagnosis of Interstitial Lung Diseases: A Series Contribution to a Debated Procedure

**DOI:** 10.3390/medicina55090606

**Published:** 2019-09-19

**Authors:** Sergio Harari, Francesca Cereda, Federico Pane, Alberto Cavazza, Nikolaos Papanikolaou, Giuseppe Pelosi, Monica Scarioni, Elisabetta Uslenghi, Maurizio Zompatori, Antonella Caminati

**Affiliations:** 1 U.O. di Pneumologia e Terapia Semi-Intensiva Respiratoria-Servizio di Fisiopatologia Respiratoria ed Emodinamica Polmonare, Ospedale San Giuseppe-MultiMedica IRCCS, via San Vittore 12, 20123 Milan, Italy; francesca.cereda@yahoo.it (F.C.); federico.pane@alice.it (F.P.); lafitta@libero.it (A.C.); 2 U.O. di Medicina Generale, Ospedale San Giuseppe-MultiMedica IRCCS, Via San Vittore, 12, 20123 Milan, Italy; 3 U.O. di Anatomia Patologica Azienda USL/IRCCS di Reggio Emilia, 42123 Reggio Emilia, Italy; alberto.cavazza@asmn.re.it; 4 Servizio Interaziendale di Anatomia Patologica, Polo Scientifico e Tecnologico, IRCCS MultiMedica, Via Gaudenzio Fantoli 16/15, 20138 Milan, Italy; nikolaos.papanikolaou@multimedica.it (N.P.); giuseppe.pelosi@multimedica.it (G.P.); 5 Dipartimento di Oncologia ed Onco-ematologia, Università degli Studi di Milano, 20122 Milan, Italy; 6 U.O. di Anestesia e Rianimazione, Ospedale San Giuseppe-MultiMedica IRCCS, Via San Vittore, 12, 20123 Milan, Italy; monica.scarioni@multimedica.it; 7 Dipartimento di Diagnostica per Immagini e U.O. di Radiologia MultiMedica IRCCS, 20123 Milan, Italy; elisabetta.uslenghi@multimedica.it (E.U.); maurizio.zompatori@multimedica.it (M.Z.); 8 Dipartimento Universitario DIMES, Università di Bologna, 40126 Bologna, Italy

**Keywords:** lung biopsy, interstitial lung disease, diagnosis

## Abstract

*Introduction*: Transbronchial cryobiopsy is an alternative to surgical biopsy for the diagnosis of fibrosing interstitial lung diseases, although the role of this relatively new method is rather controversial. Aim of this study is to evaluate the diagnostic performance and the safety of transbronchial cryobiopsy in patients with fibrosing interstitial lung disease. *Materials and methods*: The population in this study included patients with interstitial lung diseases who underwent cryobiopsy from May 2015 to May 2018 at the Division of Pneumology of San Giuseppe Hospital in Milan and who were retrospectively studied. All cryobiopsy procedures were performed under fluoroscopic guidance using a flexible video bronchoscope and an endobronchial blocking system in the operating room with patients under general anaesthesia. The diagnostic performance and safety of the procedure were assessed. The main complications evaluated were endobronchial bleeding and pneumothorax. All cases were studied with a multidisciplinary approach, before and after cryobiopsy. *Results*: Seventy-three patients were admitted to this study. A specific diagnosis was reached in 64 cases, with a diagnostic sensitivity of 88%; 5 cases (7%) were considered inadequate, 4 cases (5%) were found to be non-diagnostic. Only one major bleeding event occurred (1.4%), while 14 patients (19%) experienced mild/moderate bleeding events while undergoing bronchoscopy; 8 cases of pneumothorax (10.9%) were reported, of which 2 (2.7%) required surgical drainage. *Conclusions*: When performed under safe conditions and in an experienced center, cryobiopsy is a procedure with limited complications having a high diagnostic yield in fibrotic interstitial lung disease.

## 1. Introduction

Interstitial lung diseases (ILD) represent a very heterogeneous group of lung disorders that sometimes has a wide spectrum of overlapping clinical and radiological features often showing several complex therapeutic and prognostic implications. Obtaining a definitive diagnosis is often very difficult and clinicians dealing with these conditions are often engaged in a long and complex process of diagnostic work-up in order to reach a reliable diagnosis that is of crucial importance before starting an appropriate medical therapy. Despite a complete medical history, physical examination, and high-resolution chest CT (HRCT), the cytological profile obtained by fiberoptic bronchoscopy with broncho-alveolar lavage (BAL) as well as serological and immunological tests, physicians are frequently unable to reach a specific diagnosis. It is at this point that the histopathological examination of the lung play a fundamental diagnostic step to be taken with the context of a multidisciplinary discussion (MDD) [1]. In patients with diffuse fibrosing ILD without a known cause and with an HRCT that shows a pattern that is different from the usual interstitial pneumonia pattern (UIP), the recently updated guidelines for the diagnosis of idiopathic pulmonary fibrosis (IPF) do in fact recommend performing a surgical biopsy of the lung [1]. Obtaining an adequate sample of lung tissue having histopathological diagnostic characteristics involves invasive procedures that are not without risks and complications. The guidelines indicate surgical lung biopsy (SLB) for those patients suspected of having IPF who are however at minimal surgical risk. The mortality rate of elective SLB is of ~1.5%; the need to obtain a definitive histopathological diagnosis must be weighed against the risks associated with the procedure [2,3]. Patients with multiple co-morbidities, poor general health or severe lung impairment are at higher risk. Practically, up to 15% of patients are left without a specific diagnosis—they fall into the category of the so-called unclassifiable interstitial diseases [1]. Patients with an atypical clinical and radiological picture who, for various reasons cannot perform an SLB, fall into this category [4]. SLB is currently considered the gold standard for obtaining a pulmonary histological sample, making it possible to reach a definitive histological diagnosis in over 90% of cases [2].

However, recent data from randomized controlled clinical trials indicate that histological information is required in at least 30–40% of patients with IPF [5,6]. In the absence of a pulmonary biopsy, the diagnosis of IPF is underestimated, since the typical radiological pattern of IPF is found only in 50% of cases, while in the remaining cases the HRCT picture is rather atypical and does not allow for a reliable diagnosis. The search for a less invasive alternative to SLB is prompted by the need to reduce the prevalence of unclassifiable ILD, lower the risk of complications associated with surgical procedures, and offer the opportunity to obtain a diagnostic biopsy lung specimen in a larger number of patients.

Until a few years ago, the alternative to thoracoscopy was the trans-bronchial biopsy (TBB). However, TBB allow the collection of very small fragments of pulmonary parenchyma (1–3 mm); the analysis of these fragments is often limited by artifacts related to the procedure itself (the so-called “crushing artifacts”), and may be inadequate to diagnose a UIP pattern, the histopathological correlate of IPF. The sensitivity of the procedure is very low. The overall diagnostic yield of TBB varies considerably, from 25% to 75%, based upon the type of lung disease [7,8]. Several studies have shown that TBB can detect a UIP pattern in 30% of cases, with high specificity and high positive predictive value, but with low negative predictive value [9,10]. Sheth et al. in their study prove that the information derived from TBB combined with clinical and radiological data (HRCT) can provide enough evidence to formulate an accurate diagnosis in about 20–30% of patients with diffuse infiltrative lung disease [10]. The diagnostic yield may even be higher than 80–90% for non-fibrosing ILDs [9,11,12]. TBB can have a diagnostic value in cases of sarcoidosis, hypersensitivity pneumonia, eosinophilic pneumonia, organizing pneumonia, carcinomatous lymphangitis, diffuse alveolar damage, amyloidosis, proteinosis, microlytiasis, and infections [7,13,14]. Therefore, the small size of the biopsy sample and the high incidence of artifacts limit the role of TBB in the diagnostic work-up of ILDs [7,15]. Less invasive methods were then considered, in the hope of providing a diagnostic test as accurate as SLB, which would allow for larger histological samples than with TBB and with a better safety profile than SLB. Transbronchial lung cryobiopsy (TBLC) would seem to be a promising and safer alternative to SLB in the diagnostic approach to fibrosing ILDs [16]. TBLC is a bronchoscopic technique where, instead of the bioptic forceps, a probe is introduced through the operative channel of the bronchoscope cooled to a very low temperature (about −80 °C) for a few seconds. The cryosurgical equipment works by exploiting the Joule-Thomson effect that freezes a fragment of the pulmonary parenchyma, which therefore remains attached to the probe and can then be retrieved [17]. Compared to conventional TBB this method enables the retrieval of a larger fragment of lung parenchyma (7–10 mm) with less artifacts, being therefore more suitable for diagnostic purposes [18]. Variable results were reported by a number of studies regarding the diagnostic yield and safety of TBLC for the diagnosis of ILD [16,19,20,21,22]. The diagnostic yield would appear to be acceptable, but doubts were raised regarding the safety of the procedure. Nevertheless, the results of TBLC have been used in MDD and the results are in favour of TBLC viewed as a viable alternative to SLB [18]. The true role of TBLC in the diagnosis of fibrotic ILD is currently not completely clear and is the subject of a lively debate. 

## 2. Aim of the Study

Aim of the study was to determine the diagnostic accuracy of TBLCs performed at the Division of Pneumology of the San Giuseppe-Multimedica Hospital in Milan for the diagnosis of fibrosing ILDs in those patients whose clinical and radiological data had proven insufficient to establish a definitive diagnosis after MDD. Further objective of the present study was to examine the safety profile of the procedure, taking into consideration the two major complications: pneumothorax and endobronchial bleeding. 

## 3. Materials and Methods

Patients enrolled in this study had an uncertain clinical and radiological diagnosis and were followed at the Division of Pneumology San Giuseppe Hospital in Milan. The clinical data of these subjects were discussed at multidisciplinary level by pulmonologists, pathologists and radiologists: patients were then scheduled to TBLC between May 2015 and May 2018. Medical history, complete physical examination and screening including autoimmunity testing were carried out in all cases. All patients, before performing TBLC, underwent anesthesiologic examination and respiratory function tests (overall spirometry and DLCO), 6-min walk test, resting arterial blood gas and transthoracic color-doppler echocardiography. The Ethical Code approval number is CE-71.2019/Pr 377.2019 and the date of approval is 23 July 2019.

**Figure 1:**
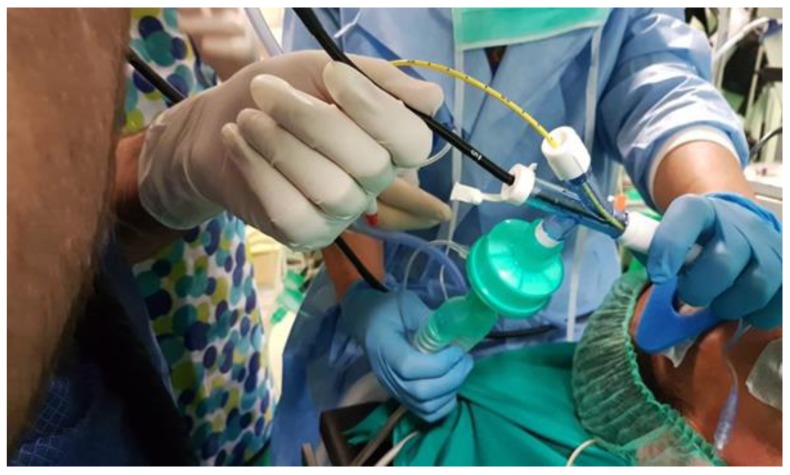
The patient is under general anaesthesia and intubated: a fibrobroncoscope and a bronchial blocker are inserted into the endotracheal tube.

Initially, in accordance with the hospital protocol, the procedure was performed in short-term hospital stay regime (lasting 2–3 days); later, due to a change in hospital organization, the duration of the stay was shortened and patients are now undergoing all the evaluations in pre-hospitalization regime. They are then admitted to hospital, the procedure is performed on the same day of admission and, if no complications occur, they will be discharged the following day. Lung tissue TBLC can be performed during flexible or rigid bronchoscopy, but we performed all the procedures with the flexible one. In our practice, patients are deeply sedated with IV propofol, with or without remifentanil, and intubated with a wire-armoured or rigid endotracheal tube. On one side, a bronchial blocker is inserted, of the same type normally used for thoracic surgery; it is wider than the Fogarty balloon and therefore it allows the closure of a lobar bronchus and the insertion of water or drugs during the procedure. Oxygen is continuously pumped through the tube. Spontaneous respiration is maintained throughout the procedure or, in case of paralysis induced by the use of non-depolarizing blocking agents, jet ventilation is used. Oxygen saturation, arterial blood pressure, electrocardiogram (ECG) and transcutaneous partial CO_2_ pressure are continuously monitored. The entire procedure is performed in the operating room. A fibrobronchoscope is inserted in the other side and through it the cryo-probe is introduced (Figure 1). The lung segment to be biopsied is identified prior to the procedure according to HRCT findings.

Only the 1.9-mm cryo-probe has been used in our center. The cryo-probe is introduced through the fibrobronchoscope under fluoroscopic guidance and brought to the periphery of the lung. Optimal conditions are considered to be a distance of approximately 10–20 mm from the chest wall and a perpendicular relationship between the chest wall and the probe. Once the correct position is reached, the probe is cooled down to −80 °C for about 3–6 s. The fragment of lung parenchyma close to the probe remains attached to the cryo-probe and can thus be retrieved. The frozen tissue attached to the tip of the probe is extracted by pulling the cryo-probe together with the bronchoscope: because the biopsy tissue is larger than the working channel of the bronchoscope and the frozen distal end of the cryo-probe could damage the working channel of the instrument during the retrieval of the biopsy. The frozen sample is thawed in saline and then fixed in formalin. At the end of the procedure, a control chest X-ray is always performed after 4–6 h from the end of the procedure. The average size of the biopsy fragments is 5 mm in diameter and usually 3–4 samples are obtained from each patient. Overall, the procedure takes approximately 20 min. The biopsy fragments are analyzed at the Pathological Anatomy of the San Giuseppe Hospital (NP, GP) under the supervision of a pathologist with expertise in reading these particular types of histological specimens (AC). Biopsies were considered “non-diagnostic” when histopathologic criteria sufficient to define a characteristic histopathologic pattern were lacking (i.e., normal lung or minimal nonspecific changes). Specimens were considered inadequate if too small or containing only airway wall with no alveolated lung parenchyma.

Pathologists provided their level of confidence in the diagnosis (high or low). The level of confidence was quite subjective: in general the combination of patchy fibrosis and fibroblastic foci corresponded to a high level of confidence for the diagnosis of UIP, whereas the presence of just patchy fibrosis, just fibroblastic foci or just honeycombing corresponded to a low level of confidence. This approach provided information to the multidisciplinary team for MDD. Knowing that cases will go through MDD reminded the pathologist that the histologic impressions could be supported (or refuted) from other information presented in the MDD. All data were retrospectively collected. 

## 4. Statistical Analysis

A descriptive analysis of the population was performed taking into account age, gender, BMI, smoking status and number of cigarette packets smoked/year. Cardiac function parameters (TAPSE and TRV assessed with echocardiogram) and pulmonary function values (spirometry data, 6MWT, arterial pO2) were also collected and analysed. Mean and standard deviation (DS) of the measured parameters were calculated. The diagnostic accuracy of cryobiopsy specimens was also evaluated; the pathologist provided a histological diagnosis wherever possible after having analyzed the specimens and indicated their suitability. To assess the safety of this procedure, the two major complications during or immediately following the procedure were considered: pneumothorax and bleeding (major or minor blood loss). Of these two events, the frequency at which they had occurred was taken into account and, as for bleeding, the difference between minor and major blood loss was established on the basis of the actions taken: simple instillation of adrenaline for minor bleeding and blood transfusion for major bleeding. This distinction was made arbitrarily by our center as yet there is no standardization to define the extent of bleeding. The cryobiopsy procedure was always performed by the same operator (SH). 

## 5. Results

This study enrolled 73 patients with a non-diagnostic clinical-radiological picture after MDD (Table 1). In the study period (May 2015–May 2018), about 350 incident patients with ILDs were followed at our hospital. These are the first patients that underwent cryobiopsy at our center. Eighteen patients (24.6%) had no comorbidities. The most frequent comorbidities observed in our series were systemic arterial hypertension (34.2%), diabetes (23.3%), gastroesophageal reflux (20.5%), cardiovascular diseases (16.4%), dysthyroidism and dyslipidemia (9.6%). Subject characteristics are summarized in Table 1.

The functional examinations show that the population undergoing cryobiopsy is composed of patients with mild/moderate disease. No pulmonary hypertension was observed; the average respiratory function was preserved even though the DLCO values remained low (with an average of 53.5%). Latent respiratory distress was observed in 17 cases (23%). None of the cases involved respiratory failure in resting conditions (Table 2).

**Figure 2:**
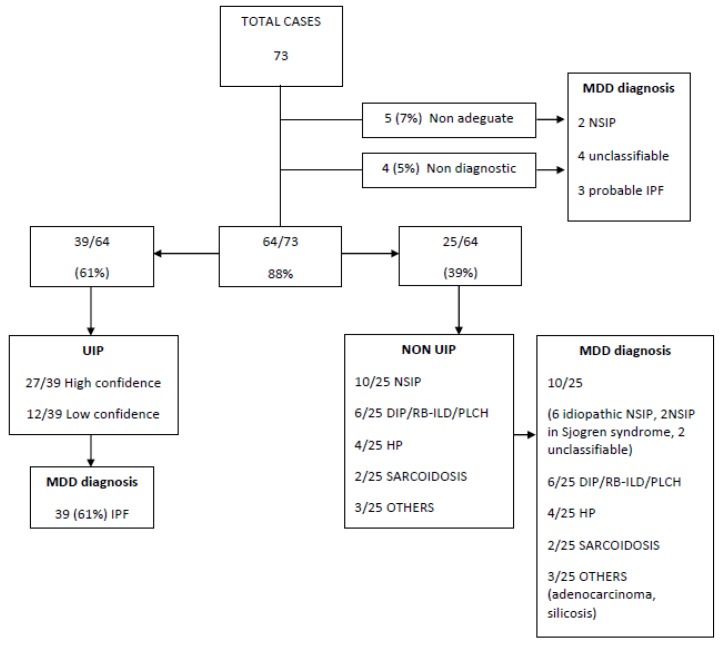
The diagnostic algorithm used for the management of our cases.

In 64 of the cases, a specific diagnosis was reached with a diagnostic sensitivity of 88%. In 5 cases, the cryobiopsies were not suitable for histological diagnosis (7%); in 4 more cases cryobiopsies did not qualify for diagnosis (5%). In 39/64 cases (61%) a pathological diagnosis of UIP with high or low confidence interval was obtained. In 25/64 cases (39%) a variety of conditions were observed, ranging from NSIP to smoking-related disorders, from chronic HP (4 cases) to sarcoidosis (2 cases); in 3 more cases, other conditions were identified, including lepidic growth adenocarcinoma. The definitive diagnoses were then established at MDD (Figure 2).

Only one case of major bleeding event (1.4%) was observed with regard to complications. This was certainly related to the learning curve of the technique (this was the second case to be performed). Fourteen patients had a minor bleeding episode (19%) resolved during the procedure after endobronchial instillation of adrenaline. Eight cases of pneumothorax (10.9%) were identified, of which only two (2.7%) required surgical drainage; however, they were resolved without any sequelae for the patient. Only one case of long-term respiratory failure was observed (the same with severe bleeding). The mean hospital length of stay was 3.61 days (min 2; max 20). No delayed complications were observed.

## 6. Discussion

The percentage of patients who underwent cryobiopsy was about 20% of all patients with ILDs observed at our hospital. In our experience with TBLC, this technique allowed us to reach a definitive diagnosis in 88% of cases with a good safety profile. Diagnostic performance was assessed by the pathologist’s ability to provide a histopathologic diagnosis, the ability to come to final diagnosis upon MDD and the patients’ follow-up. The gold standard for the final diagnosis of ILD is actually considered a multidisciplinary discussion: we therefore used this as reference. The average age of our population reflects the age of the population with IPF (the disease most frequently reported in our series). These are the first patients who underwent cryobiopsy at our center; this explains the limited severity of the functional impairment of our population and the few comorbidities. Most patients with ILDs do not need a histological diagnosis and can therefore be simply classified on the basis of clinical and radiological data, thus avoiding invasive procedures. However, the percentage of agreement among radiologists, even during MDD, may not be very high concerning the individual interpretation of specific clinical cases [1,7,23]. At the same time, there is a large proportion of patients who, despite our attempts, cannot be classified simply on the basis of clinical-radiological data [1]. All this leads us to state that histology still plays an important role, especially in the more complex and difficult cases. Surgical biopsy represents the gold standard [1]. However, we know that every surgical procedure carries a certain risk of morbidity and mortality, especially in patients with advanced pathology and can, although rarely, trigger acute exacerbations, particularly in patients with IPF [24,25,26,27]. The risk–benefit assessment of a diagnostic procedure is therefore essential, bearing in mind that therapeutic relapses sometimes do not significantly change the clinical history of patients. Furthermore, one should not forget the patient’s personal choice: in some cases, it is the patient himself who refuses a surgical operation for purely diagnostic purposes. Therefore, each patient must be carefully evaluated and a decision taken as to what the invasive diagnostic procedure can offer more in terms of diagnostic and therapeutic benefit. For several reasons, surgical biopsies have so far been limited to a fairly small number of patients (about 20% of patients with ILD) [10,28]. TBLC represents an increasingly recognized method for obtaining samples of lung parenchyma, even in complex fibrosing ILDs [18,29,30]. Cryobiopsy has the advantage of being relatively safe, with a limited percentage of complications and a much lower mortality rate compared to SLB [18,29,30]; it also has a diagnostic yield exceeding 80%, especially when samples are taken from different lung segments [31]. Our results agree with these studies: the proportion of diagnoses is very high (88%) and competitive with SLB (90% of cases). The incidence of complications is significantly lower: in our study we notice a 10.9% incidence of pneumothorax: a remarkably low value if we consider that pneumothorax occurs in 100% of cases of surgical biopsy with VATS, since pneumothorax is mandatory to carry out the procedure. In regards to bleeding, the incidence of major bleeding episodes with cryobiopsy that we have observed was 1.4% versus 13% for surgical biopsy [2,28,32,33]. 

In our study, 39 patients had a histological diagnosis of UIP pattern and a subsequent diagnosis of IPF after MDD of the case and could therefore have access to the new antifibrotic drugs (pirfenidone and nintedanib), which they would not have been able to take without performing the cryobiopsy, since the HRCT findings were not conclusive. Therefore, although surgical biopsy is still the gold standard for histological sampling in fibrosing ILDs, numerous studies suggest cryobiopsy is just as valid, almost equivalent and a less dangerous alternative due to its lower risk of morbidity and mortality [18,19,20,22,29,30,34,35]. Cryobiopsy is not as yet recommended by the current guidelines due to the absence of conclusive evidence, including the lack of studies directly comparing cryobiopsy with SLB to finally validate this procedure for the diagnostic work-up of fibrosing ILDs [1,16]. The pathological analysis is a crucial step in order to increase the diagnostic accuracy of the procedure and to validate its use in clinical practice [36]. The pathologist should analyze the TLCB specimens using the same criteria as those accepted for specimens obtained from SLB. The international increase in the use of TLCB calls for a precise standardization of the analytical and reporting methods used by pathologists for the specimens obtained with TLCB [37]. Romagnoli et al. highlight a low correlation between the two procedures [38]. The only way to effectively determine the diagnostic accuracy of cryobiopsy is to perform both procedures on the same patient and then report the results in an MDD. Cryobiopsy and surgical biopsy during VATS proved to be correlated in only 38% of cases (95% CI: 18–62). Cryobiopsies proved to be non-diagnostic in 19% of cases, while no SLB specimen resulted to be non-diagnostic. The K coefficient of concordance between the two techniques was low (0.22; 95% CI: 0.01–0.44) [38]. The diagnostic performance of the two procedures is not necessarily identical and the diagnosis of IPF is more accurate with SLB [39]. 

In conclusion, this study, which is currently unique in its kind using this technique [38], shows that the diagnostic yield of the two procedures is different and that the outcome of the SLB more often matches the final diagnosis obtained during MDD. Therefore, SLB can be currently considered as the procedure of choice when a histological assessment is required. This evidence is however not surprising: it seems logical to assume that the histopathology of larger biopsy specimens obtained from the periphery of the lung can be more reliable. Another very plausible explanation for this low reproducibility is that the diagnostic accuracy of the procedure is highly dependent on the location of the biopsies, the size of the specimens and the degree of patchy inhomogeneity of pathological changes in the lungs [16]. This data seems to produce evidence that goes against the routine use of cryobiopsy in the clinical practice for ILDs; in reality, the procedure, the sampling technique and preservation method of an adequate number of specimens, the interpretation criteria of pathologist, need to be better defined in order to achieve a high level of standardization as in any new procedure before it is introduced in clinical practice [36]. Although according to many studies, cryobiopsy may appear to be less accurate than surgical biopsy, it could be argued that, being safer, it may be an acceptable diagnostic step in many cases, especially for patients at high surgical risk. The work of Romagnoli et al. sparked a lively debate about the role of this new technique and an impressive number of letter and comment have been recently published [40,41,42,43,44,45,46,47]. While in our study, the results concerning the safety of the procedure appear to be reassuring, other investigations have revealed a higher complication rate more specifically related to bleeding, especially in the less experienced centers [48]. The safety and efficacy of the procedure must be better defined, possibly through future prospective trials. 

Our study has many limitations: first) it is a retrospective and monocentric study; second, there is no control group and diagnostic confirmation with SLB; third, the number of patients is small. However, this is a relatively new technique and our initial use of this procedure confirms the results about its diagnostic accuracy and safety.

## 7. Conclusions

Although SLB still remains the gold standard, where histological examination of lung parenchyma is considered necessary from a diagnostic standpoint after MDD, various scientific evidence suggests that cryobiopsy is a less dangerous and almost equivalent alternative to SLB. The debate about the real role of TBCB need to be further define. Cryobiopsy is a very interesting procedure, yielding satisfactory results from a diagnostic standpoint. If performed safely and in an experienced center, cryobiopsy has a high diagnostic yield with limited complications in the area of ILDs. The indication should be properly evaluated, and therefore performed in those patients who may have diagnostic benefit. Italy is a country at the forefront at European level, with a considerable number of cases in the implementation of this diagnostic procedure. It must however be carried out in total safety, because we must not forget its diagnostic nature: it would be unacceptable to risk serious consequences during a diagnostic test. It is not a simple technique and it must therefore be carried out in expert centers, which can achieve a certain volume of activity. This is the only way to improve the technical expertise of both the operator and the pathologist of the centers, reduce complications and improve the diagnostic performance. Cryobiopsy still has no defined standards, such as the freezing time and the discrimination between major and minor bleeding, also because only about 1000 cryobiopsies have been performed worldwide. As cases increase in number, this technique will undoubtedly be properly standardized and, perhaps, included in the new guidelines for the diagnosis of fibrosing ILD. This technique has the advantage of allowing a histological diagnosis in a much wider number of patients; this means that we can offer new treatment options, such as the new antifibrotics, to patients with IPF who would not otherwise be diagnosed.

## Figures and Tables

**Table 1:** Baseline clinical characteristics of patients.

Mean ± SD	
Age (years)	66.6 ± 8.6
BMI	27.0 ± 4.2
Gender (F/M)	28 (38%)/45 (62%)
Smoking status (ex/current/never)	39/9/25 54%/12%/34%
Pack/year smoked (mean)	28.7

Abbreviations: SD: standard deviation; BMI: body mass index, F: female, M: male.

**Table 2:** Baseline cardio-pulmonary function.

	Mean ± SD
FEV1 (L)	2.19 ± 0.6
FEV1 (%)	83.0 ± 17.1
FVC (L)	2.63 ± 0.8
FVC (%)	80.6 ± 18.3
TI (%)	81.5
TLC (L)	5.09 ± 1.6
TLC (%)	85.2 ± 22.6
DLCO (mL/mmHg/min)	12.9 ± 4.4
DLCO (%)	53.5 ± 15.5
6MWT on RA (m)	449 ± 103
Desaturation rate (pt%)	4.5 ± 5
Exertional respiratory failure, *n* (%)	17/73 (23%)
TAPSE (mm)	24 ± 4,1
TRV (m/sec)	2.73 ± 0.42
pO2 on RA (mmHg)	81.1 ± 8.3

Abbreviations: SD: standard deviation; FEV1: force expiratory volume in the 1st second; L: liter; FVC: forced vital capacity; TI: index of Tiffenau; TLC: total lung capacity; DLCO: diffusing capacity of the lungs for carbon monoxide; 6MWD: six-minute walking distance; RA: room air; m: meters; pt: point; *n*: number; TAPSE: tricuspid annular plane systolic excursion; mm: millimeter; TRV: tricuspid regurgitant velocity.
